# Recurrence of diffuse large B-cell lymphoma in sciatic and tibial nerves: A case report

**DOI:** 10.1016/j.radcr.2023.10.063

**Published:** 2023-11-22

**Authors:** Hourieh Soleimani, Farzaneh Khoroushi, Sajad AtaeiAzimi, AmirHossein Jafarian, Omid Salarzaei, Behzad Aminzadeh

**Affiliations:** a Department of Radiology, Faculty of Medicine, Mashhad University of Medical Sciences, Mashhad, Iran; b Department of Hematology-Oncology, Mashhad University of Medical Sciences, Mashhad, Iran; c Department of Pathology, Pathology Cancer Molecular Research Center, Mashhad University of Medical Sciences, Mashhad, Iran; d Faculty of Medicine, Mashhad University of Medical Sciences, Mashhad, Iran

**Keywords:** Neurolymphomatosis, B-Cell Lymphoma, Neuropathy, Level of evidence 4

## Abstract

Infiltration of peripheral or cranial nerves with lymphatic cells is a rare condition that is known as neurolymphomatosis (NL). The involvement could be primary or secondary and mostly occurs in patients with a history of B-cell lymphoma. The most common peripheral nerve involved is the sciatic nerve. Patients may present with painful or painless mononeuropathy or polyneuropathy, and MRI is the perfect modality to evaluate the suspicious clinical findings that may demonstrate enlargement, thickening, and enhancement of the involved nerve or an enhancing mass lesion in the course of the nerve. Biopsy can be safely performed to confirm the diagnosis. Few articles have reported the cases of peripheral nerve involvement by lymphoma as well as MRI features of this diagnosis. In this article, we report a case of NL using MRI, ultrasound, and pathologic features and also present a brief review of relevant literature.

## Introduction

Lymphatic infiltration of peripheral nerves, also known as neurolymphomatosis (NL), is an extremely rare condition in patients with known systemic involvement. In fact, the disease may relapse or show recurrence in the nervous tissue [1]. It could be also the first manifestation of non-Hodgkin`s lymphoma in rare cases, which is called primary neurolymphomatosis. Peripheral nerve involvement may manifest as solitary or multifocal lesions and sciatic nerve is the most common site for lymphoma involvement [2]. In addition, cranial nerves or spinal roots may be involved by lymphatic infiltration [3,4]. NL occurs in 5% of patients with diffuse large B-cell lymphoma (DLBCL) [4]. NL is a distinguishable entity from the compression of a nerve by lymphadenopathy and it is also different from CSF seeding of primary or secondary CNS lymphoma, which is called meningeal lymphomatosis [5]. Patients most commonly present with painful polyradiculopathy; however, other presentations like palpable mass lesions or foot drops may occur [6,7]. These symptoms are not specific for NL and have a lengthy list of differential diagnoses but in the setting of known previous lymphoma may direct the clinician toward neurolymphomatosis as a probable diagnosis.

In this paper, we report a case of NL. A 69-year-old male who had a history of DLBCL and was in the remission phase following chemotherapy showed a relapse of DLBCL in the distal sciatic nerve and proximal tibial nerve.

## Case report

A 69-year-old man with a history of DLBCL diagnosed in January 2021, presented to the hematology clinic with swelling in the left popliteal fossa, neuropathic pain, and weakness. The first manifestation was a nasopharyngeal mass lesion; chemotherapy began at that time of hospitalization and the patient was treated with Docetaxel (Zytax), vincristine, Adriamycin, dexamethasone as well as intravenous and intrathecal methotrexate. Also, he had a history of scalp squamous cell carcinoma (SCC) 10 years ago. The patient was referred 7 months later in October 2021 with a Left side popliteal palpable mass and neuropathic pain.

**Fig. 1:**
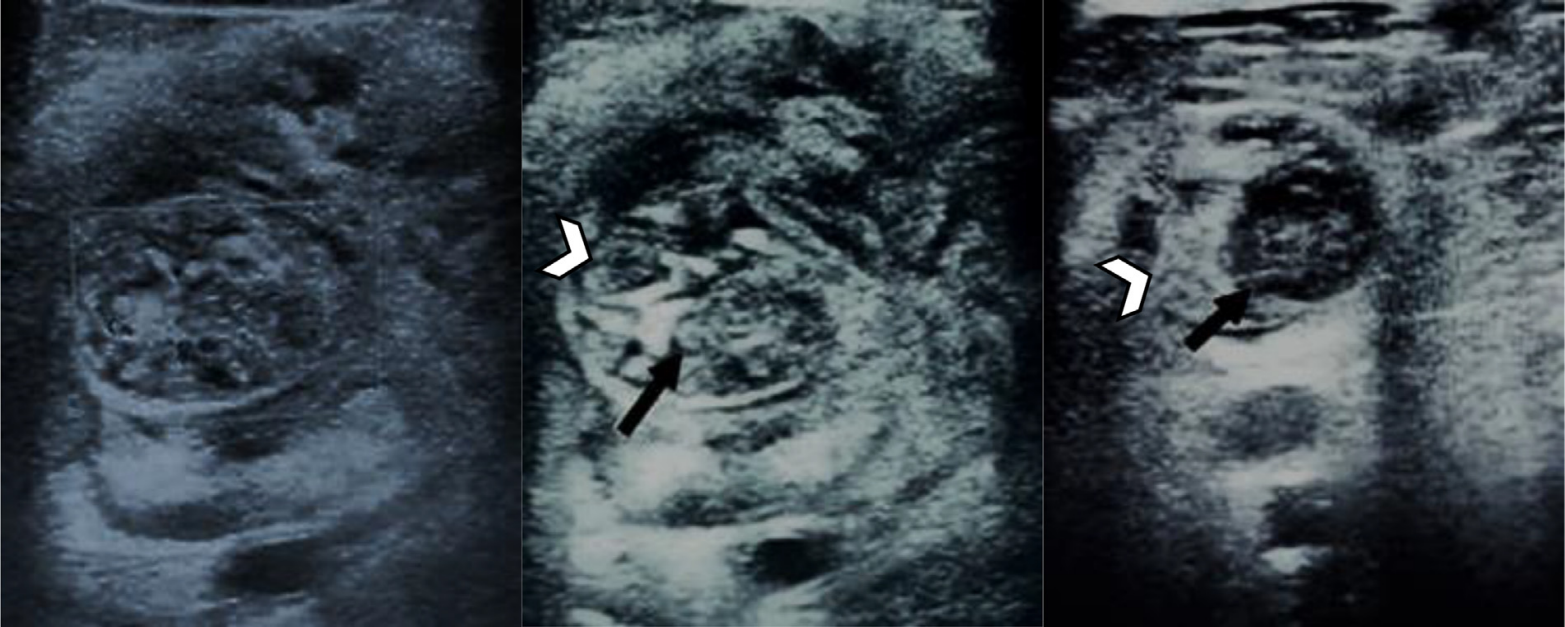
Ultrasound images of the left popliteal fossa demonstrated an enlarged and infiltrated nerve with honeycomb appearance (arrows) surrounded by a mixed echogenicity mass (arrowheads).

Ultrasonography of the left popliteal fossa demonstrated a heterogeneous echogenicity mass with internal vascularity in the popliteal fossa and sciatic and tibial nerves as a cable-like edematous structure within the mass (Fig. 1). Various differential diagnosis has been considered for a painful popliteal mass in an elderly patient, such as soft tissue sarcoma, lymphadenopathy, and benign or malignant primary peripheral nerve sheath tumors. Clinical presentation of neuropathic pain with a previous history of lymphoma, as well the ultrasound findings of an edematous cable-like structure accordant to the pathway of sciatic and tibial nerves which pass through the mass and being infiltrated by the lesion, not only displaced with it, suggested that neoplastic infiltration of the nerves sheath could be the first diagnosis. Therefore, an MRI of the left knee was performed for further evaluation.

**Fig. 2:**
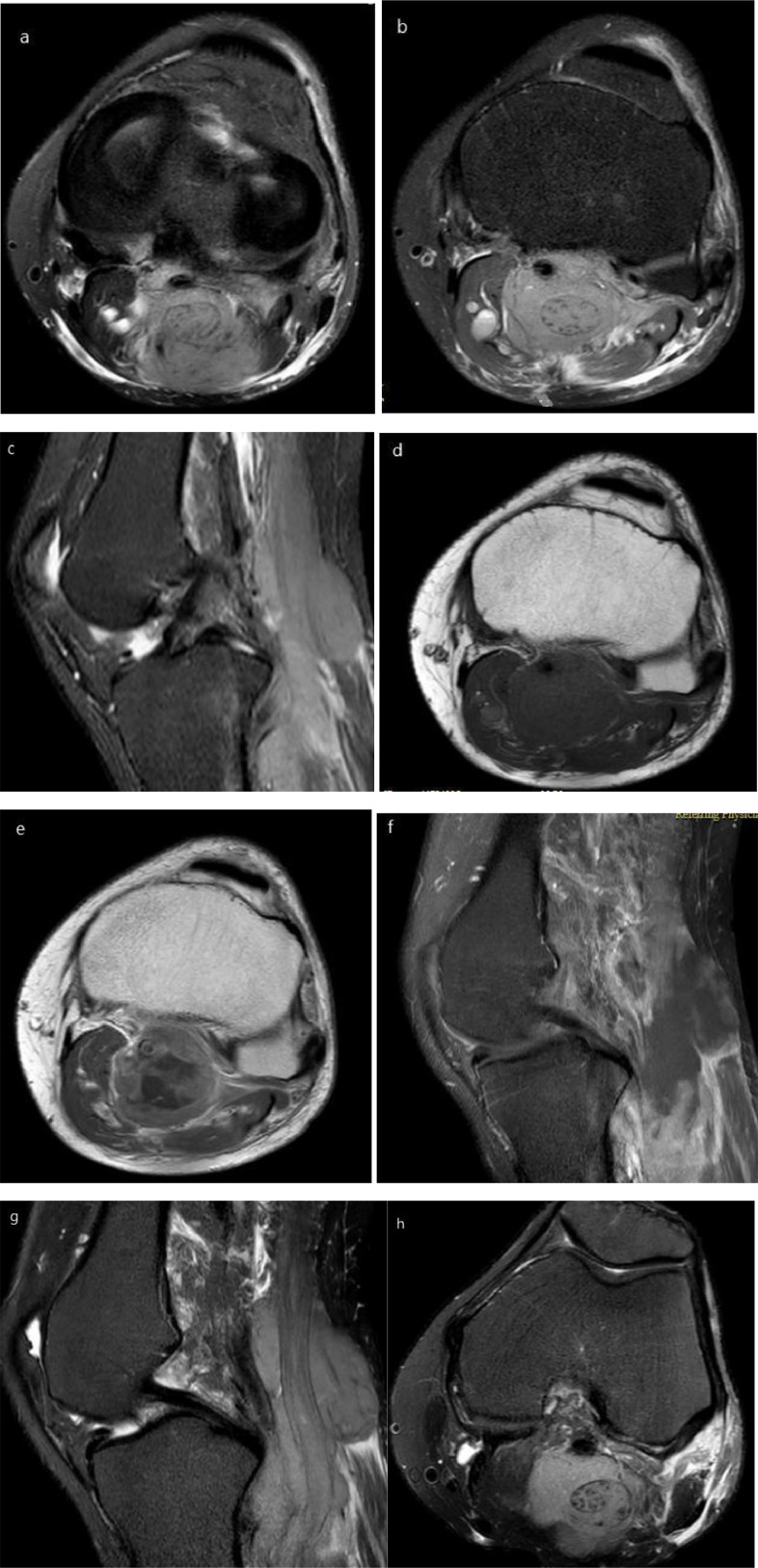
MRI of the left knee. A–H. (A) Axial T2; (B) Axial proton density fat-saturated; (C) sagittal STIR; (D) axial T1-weighted; (E) axial postcontrast T1-weighted; (F) sagittal postcontrast T1-weighted fat-saturated; (G) sagittal T2-weighted fat-saturated and (H) axial T2-weighted fat-saturated. Selected MRI images demonstrated a large fusiform mass with heterogeneous high signal intensity in T2-weighted and intermediate signal intensity in T1-weighted along the course of tibial nerve, which is enlarged and infiltrated. Heterogeneous predominantly peripheral enhancement is seen in postcontrast T1-weighted images.

**Fig. 3:**
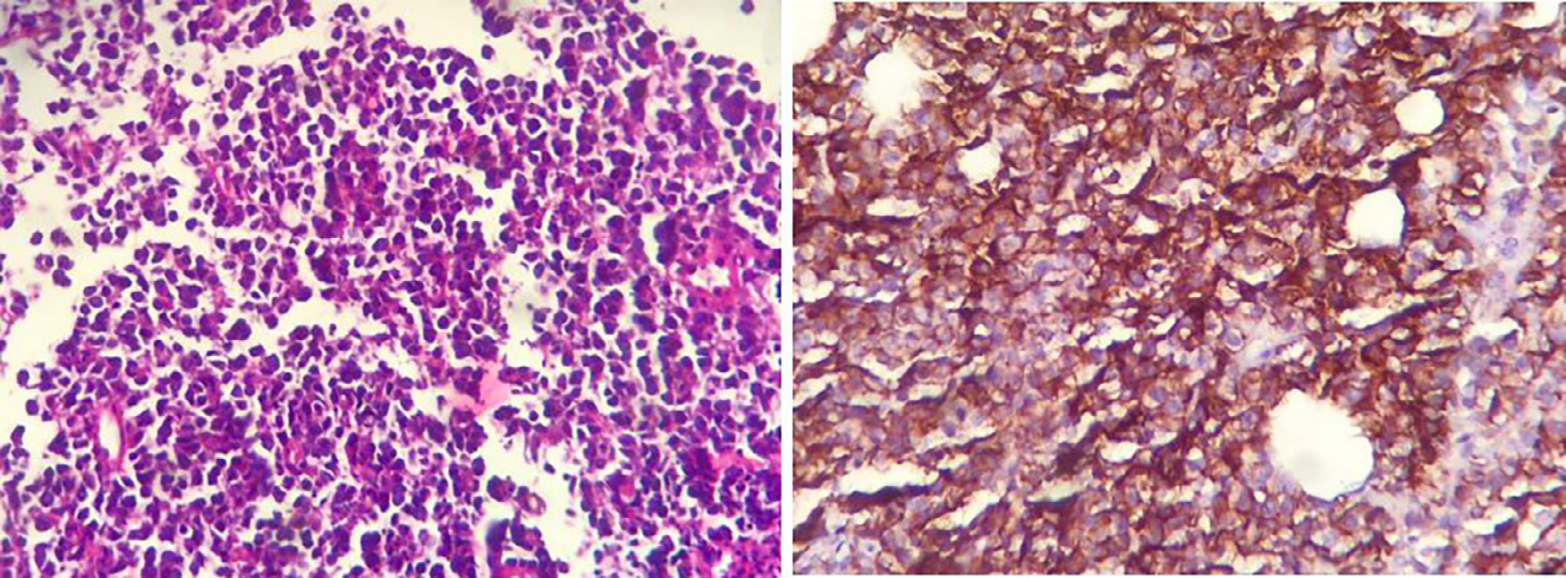
(A and B) High power magnification shows diffuse infiltration of large atypical lymphoma cells with amphophilic cytoplasm and occasional prominent nucleoli, and CD20 staining is positive in neoplastic cells and identifies the B-cell nature.

MRI demonstrated a fusiform infiltrating mass lesion measuring 83 × 44 × 35 mm with intermediate signal intensity in T1-weighted, high signal intensity in T2-weighted, and STIR images with heterogeneous predominantly peripheral enhancement in the popliteal fossa. Perilesional edema and encasement of popliteal artery in a part of its pathway were seen. Distal sciatic and proximal tibial nerves were infiltrated by the mass, were enlarged, and passed through the mass, and were involved by it and is described as “Entering and exiting nerve sign” (Fig. 2). Ultrasound-guided core needle biopsy was done and the neurolymphomatosis was confirmed. Histologically, this neoplastic lesion is composed of diffuse infiltration of large atypical cells with amphophilic cytoplasm and occasional prominent nuclei resembling immunoblasts or centroblasts (Fig. 3). Treatment started with GCD-R chemotherapy protocol (gemcitabine, carboplatin, dexamethasone, and rituximab). A short-term follow-up showed improvement of the irritating symptoms and also decreased size of the mass lesion.

## Discussion

Lymphoma is defined as a neoplastic proliferation of lymphoid cells from a secondary lymphoid structure, a lymph node or an extranodal structure. It is well established that extranodal site involvement is present in up to 40% of DLBCLs, and virtually any extranodal location can be a primary site [2]. However, peripheral nerve involvement in lymphomas is a rare manifestation, whether as an initial or recurrent presentation. Therefore, more information is required to improve recognition of the clinical and imaging characteristics of this uncommon presentation of DLBCLs. Neurolymphomatosis is a rare manifestation of lymphoma. There are a few articles that have reported one or more cases of NL, some of which have described related MRI and pathologic features. A majority of cases occur in the setting of diffuse lymphoma as a recurrence or relapse of the previous known disease but it is rarely the primary source of involvement [2]. The most common histologic type of NL is B-cell lymphoma with DLBCL as the most prevalent type; however, NL is occasionally observed with T-cell or NK-cell lymphoma [8,9]. Patients may be referred with neuropathic symptoms such as sciatic pain [1], numbness in the toes or limb paresthesia [2], foot drop in 1 case [7], or a case similar to ours with a palpable swelling or mass [10]. Guillain-Barré syndrome has been reported in the literature as well [11].

The most common site for involvement is the sciatic nerve but other sites have also been described in the previous case reports such as brachial[5], ulnar[12], radial, [13] and median nerves [14].

MRI is the best modality for detecting the spread of the lesion and involved nerves as well as demonstrating the relationship between the mass lesion and peripheral nerves to determine the origin of the mass [10]. Also, this modality has a sensitivity of 77% for the detection of the lesion but it is not specific enough [1,10]. Other peripheral nerve sheath tumors are in differential diagnoses such as schwannoma, perineurioma, and malignant peripheral nerve sheath tumors [1,10]. MRI features of NL described in other case reports include nodular thickening of the nerve [1], large size of the tumor, fusiform appearance, as well as predominantly peripheral enhancement of the mass in postcontrast MRI images [10]. Similarly, the case we reported showed a large fusiform mass infiltrating a peripheral nerve that was predominantly enhanced in postcontrast T1-weighted images. A case series published in July 2020 reported non-neurogenic peripheral nerve malignancy (PNM) for 5 years up to 2019. The results showed that approximately 7% of PNMs are non-neurogenic, that metastasis is the most common cause (63%), and that the most common site is lumbosacral plexus/sciatic nerve. A helpful MRI feature for the differentiation of neurogenic from non-neurogenic PNMs is the absence of target signs as well as other typical signs frequently observed in neurogenic tumors; DWI images or FDG-PET are also used for differentiation [15].

Another case series published in 2016 reported 5 patients with peripheral and cranial NL during 18 months, all of whom had a history of previous lymphoma diagnosis. Their results showed that enlargement, thickening, and enhancement of nerves were the most frequent appearances of NL in MRI images [8]. Another finding in Australia reported 3 patients with NL diagnosed by MRI and 18FDG-PET scan having involvement of sciatic nerve, one of whom showed spinal nerve root involvement. Similar to our case, MRI demonstrated an enhancing mass in the course of the sciatic nerve. Intense 18FDG uptake confirmed the diagnosis but 1 patient needed pathologic confirmation that was safely done like our case report [1]. The best treatment modality for NL is controversial, and the most common chemotherapeutic agent is methotrexate in high-dose with or without other agents [16]. A literature review showed that a high dose of methotrexate has limited efficacy in the treatment of patients with NL [16]. Other treatments explained in papers include radiotherapy together with chemotherapy and high-dose chemotherapy followed by autologous stem cell transplantation (HDC-ASCT) [3,9]; however, treatment of NL is individualized and could be different for each patient depending on the type of lymphoma, histologic findings and presentation in the time of diagnosis [9].

## Conclusion

We reported a case with a known history of DLBL who had a relapse of the disease in the distal sciatic and proximal tibia nerves. It is an uncommon presentation of lymphoma. The diagnosis of neurolymphomatosis should be considered in patients with the same history presenting with a palpable mass lesion and neuropathic pain or radiculopathy. Diagnostic imaging modalities such as MRI could be helpful in detecting a peripheral nerve mass and characterizing the lesion. Biopsy is also safe and confirms the diagnosis when it is uncertain [1,2].

## Patient consent

The authors have obtained written informed consent for publication from the patient.
